# Berberine Protects against Palmitate-Induced Endothelial Dysfunction: Involvements of Upregulation of AMPK and eNOS and Downregulation of NOX4

**DOI:** 10.1155/2013/260464

**Published:** 2013-12-09

**Authors:** Ming Zhang, Chun-Mei Wang, Jing Li, Zhao-Jie Meng, Sheng-Nan Wei, Ji Li, Richard Bucala, Yu-Lin Li, Li Chen

**Affiliations:** ^1^Key Laboratory of Pathobiology, Department of Pharmacology, Ministry of Education, College of Basic Medicine, Jilin University, Changchun, Jilin 130021, China; ^2^Department of Pharmacology, Pharmaceutical College of Beihua University, Jilin City, Jilin 132013, China; ^3^Department of Internal Medicine, Yale University School of Medicine, New Haven, CT 06520-8056, USA

## Abstract

Endothelial dysfunction is a critical factor during the initiation of cardiovascular complications in diabetes. Berberine can ameliorate endothelial dysfunction induced by diabetes. However, the underlying mechanisms remain unclear. The aim of this study was to investigate the protective effect and mechanism of berberine on palmitate-induced endothelial dysfunction in human umbilical vein endothelial cells (HUVECs). The cell viability of HUVECs was determined by MTT assays. Nitric oxide (NO) level and production of reactive oxygen species (ROS) were determined in supernatants or in the cultured HUVECs. The mRNA level of endothelial nitric oxide synthase (eNOS) was measured by RT-PCR, and the protein levels of eNOS, p-eNOS, Akt, p-Akt, AMPK, p-AMPK, and NADPH oxidase (NOX4) were analyzed. The results demonstrated that berberine significantly elevated NO levels and reduced the production of ROS. The expressions of eNOS were significantly increased, while NOX4 protein expression was decreased in berberine-treated HUVECs. Moreover, berberine upregulated the protein expression of AMPK and p-AMPK in palmitate-treated HUVECs, but had no effect on the levels of Akt. Therefore, berberine ameliorates palmitate-induced endothelial dysfunction by upregulating eNOS expression and downregulating expression of NOX4. This regulatory effect of berberine may be related to the activation of AMPK.

## 1. Introduction

Cardiovascular complications are main causes of high mortality and morbidity induced by obesity, diabetes, and metabolic syndrome. Endothelial dysfunction has been known as a critical factor and main pathological change during the development of vascular complication [1]. Lipid metabolic disorder plays a vital role in the pathogenesis of endothelial dysfunction in obesity, insulin resistance, and diabetes. An abnormality in patients with all of these disorders is an increase in the plasma concentration of free fatty acids (FFA) [2]. Elevated FFA may cause a series of pathophysiological changes in the endothelium, including endothelial nitric oxide synthase (eNOS) uncoupling, intracellular accumulation of reactive oxygen species (ROS), and cell apoptosis, which in turn contribute to accelerating the endothelium dysfunction associated with excessive acceleration of atherosclerosis. Studies showed that high concentration of FFA impair the eNOS activity and reduce the production and bioactivity of NO in endothelial cells. FFA overload attenuates Ca^2+^ signaling and eNOS activity, reduces NO production, and indirectly leads to endothelial dysfunction in endothelial cells [1]. Ye-rong found that elevated FFA could inhibit eNOS phosphorylation and its gene expression, decrease endothelium-derived NO production, and thus lead to an impairment of vasodilation in metabolic syndrome [3]. Moreover, FFA-induced endothelium dysfunction is related to the activity of NADPH oxidase, the most important enzyme for the production of O_2_^−^_,_ within the vascular wall. As O_2_^−^ inactivates NO to form peroxynitrite (ONOO^−^), it triggers a series of harmful events such as decreasing NO bioavailability, reducing the production of NO, and causing impaired vasodilatation [4]. Inoguchi et al. reported high glucose level and FFA (palmitate) stimulate ROS production through PKC-dependent activation of NAD(P)H oxidase in cultured aortic smooth muscle cells and endothelial cells, which in part accounted for the excessive acceleration of atherosclerosis in patients with insulin resistance and diabetes [5]. Elevated FFAs not only inhibit the eNOS/NO signal pathway and decrease NO production, but also activate NADPH oxidase, increase production of O_2_^−^, and reduce NO bioactivity during the development of atherosclerosis and thrombosis in vascular complications associated with obesity and diabetes. As matter of relevance, it also has been established that impaired eNOS activity upon palmitate stimulation may be linked to toll like receptor 4 (TLR4) signaling, which is a critical mediator of palmitate-induced IKK*β* and NF-*κ*B activation, and subsequent decreases in insulin signaling and NO production in endothelial cells [6, 7].

Decreasing lipotoxicity may be a key component to prevent and treat cardiovascular complications of metabolic syndrome. *Rhizoma Coptidis *(root of *Coptis chinensis *from Ranunculaceae) has been used in traditional Chinese medicine for more than 1000 years. Berberine, an isoquinoline alkaloid, a major active component of *Rhizoma Coptidis *[8], has been well reported with pleiotropic pharmacological activities, including antibacterial, antibiotic, anti-inflammatory, and antioxidant properties, as well as ameliorating effects on hyperlipidemia and hyperglycemia. Recently, both animal and clinical studies have demonstrated that berberine improves insulin resistance, decreases blood glucose levels, regulates lipid metabolism, and inhibits the progression of obesity and diabetes [8–11]. Whether berberine can improve endothelium dysfunction and prevent the cardiovascular complications associated with these disorders causes a great interest to researchers. Tang et al. reported that berberine had antioxidant effects and could increase the protective effect on diabetic complications [12]. Hao et al. demonstrated that berberine ameliorates diabetic microendothelial injury induced by the combination of high glucose and advance glycation end products *in vitro *[13]. Our previous study indicated that berberine not only modulates glucose and lipid metabolism but also ameliorates endothelial dysfunction in diabetic rats induced by high fat diet combined with streptozotocin injection. However, the underlying mechanism through which berberine improves the endothelial dysfunction to prevent the vascular complications in obesity and diabetes mellitus is still unclear. There has been no report about the effect of berberine on the lipotoxicity in the endothelium dysfunction yet. Therefore, the present study was to elucidate the protective effects and underlying mechanism of berberine on endothelial dysfunction induced by high doses of palmitate, which could provide evidence for berberine's clinical applications in the future.

## 2. Materials and Methods

### 2.1. Materials

Human umbilical vein endothelial cells (HUVECs) were obtained from American Type Culture Collection (ATCC, USA). RPIM-1640 medium and other culture reagents were obtained from Gibco Life Technologies (Gibco, Grand Island, NY, USA). Berberine was kindly provided by Northeast General Pharmaceutical Factory (Changchun, China). Palmitate, N^G^-nitro-L-arginine (L-NA), 2,7-dichlorodihydrofluorescein diacetate (DCHF-DA), and thiazolyl blue (MTT) were purchased from Sigma (Sigma Aldrich, St. Louis, MO, USA). Bovine serum albumin (BSA, fatty acid free) was purchased from Wako pure chemical industries (Japan). Kit for measuring NO was provided from Nanjing Jiancheng Chemical Factory (Nanjing, China). eNOS primers were synthesized by Lianxing Biotechnology (Dalian, China). Polyclonal antibodies of eNOS, Akt, AMPK, and NOX4 were purchased from Santa Cruz Biotechnology (Santa Cruz Biotechnology, CA). Chemical agents for western blot and RT-PCR were obtained from Sigma Aldrich. All other chemical reagents were purchased from commercial source.

### 2.2. Cell Culture

HUVECs were cultured in RPIM-1640 medium supplemented with 10% fetal bovine serum (FBS) and passaged according to the recommended procedures of ATCC. Cells in passages 4–8 were used for experiments; cells were exposed to exogenous free fatty acid (0.5 mmol/L palmitate) for 12 h or 24 h treated with or without different concentrations of berberine (1.25, 2.5 and 5 *μ*mol/L).

### 2.3. Preparation of Free Fatty Acid-Albumin Complexes

Lipid-containing media were prepared by conjugation of FFA to BSA using a modified method described [14]. Briefly, palmitate was dissolved in 0.1 M NaOH solution in 70°C water bath for fully dissolving. Then the 0.1 M sodium palmitate was mixed with 5% fatty acid-free BSA at 1 : 9 ratio and left for one hour in 37°C incubator. 10 mM palmitate stock solution was stored at −4°C. Before experiment, the stock solution was diluted in the complete culture medium to the required concentration, adjusted to a pH value of 7.5, and filter sterilized. The control solution containing fatty acid-free BSA was prepared in the same way.

### 2.4. Cell Viability Assays

The viability of the HUVECs was determined by MTT assays. Briefly, cells were plated for 24 h in a 96-well plate at a density of 1 × 10^4^ cells per well in 200 *μ*L medium. When cells grew to 60% to 70% confluence, the medium was changed to one containing 2% FBS and 0.5 mmol/L palmitate (sovled in 4.5% free fatty acid free BSA) or treated with different concentrations of berberine (1.25, 2.5, and 5 *μ*mol/L). Each treatment was repeated in 6 wells. The cells were incubated for 20 h at 37°C in a humidified chamber. MTT reagent (20 *μ*L, 5 mg/mL in PBS) was added to each well and incubated for 4 h. The microplate containing the cells was centrifuged at 1,800 rpm for 5 min at 4°C. The MTT solution was removed from the wells by aspiration. The formazan crystals were dissolved in 150 *μ*L DMSO. Absorbance was recorded at 570 nm wavelength using a Microplate Reader. Cell viability was calculated as follows: cell viability (100%) = absorbance of experiment group/absorbance of control group × 100%.

### 2.5. Measurement of NO Level

HUVECs were grown in 96-well dishes. When cells grew to 60% confluence, the medium was changed to one containing 2% FBS. The cells were treated with 0.5 mmol/L palmitate and various concentrations of berberine dissolved in ethanol for 24 h. The same volume of ethanol was included in each control group. NO release in cultured supernatants was determined by the Griess method [15].

### 2.6. Measurement of ROS Level

HUVECs were plated in 24-well dishes at a density of 6 × 10^4^ cells per well in 500 *μ*L of complete medium. When cells grew to 60% confluence, the medium was changed to one containing 2% FBS. The cells were treated with 0.5 mmol/L palmitate and various concentrations of berberine for 12 h. 2,7-Dichlorodihydrofluorescein diacetate (DCFH-DA, 10 *μ*M, Sigma) staining was employed for ROS analysis as described previously [16].

### 2.7. RNA Extraction and Semiquantitative RT-PCR

Total RNA was extracted from HUVECs using Trizol reagent (Invitrogen). RNA samples were quantified by spectrophotometry, and the integrity was assured by 1.5% agarose gel electrophoresis and ethidium bromide staining. The first-strand cDNAs were synthesized from 5 g total RNA, using SuperScript reverse transcriptase and oligo deoxythymidine primers. The reverse transcription products were amplified by PCR, using Taq DNA polymerase and specific primers for Human eNOS (forward: 5′-GTGATGGCGAAGCGAGTGAAG-3′; reverse: 5′-CCGAGCCCGAACACACAGAAC-3′, 422 bp) and glyceraldehyde-3-phosphate dehydrogenase (GAPDH, forward: 5′-CCATGGAGAAGGCTGGG-3′; reverse: 5′-CAAAGTTGTCATGGATGACC-3′, 194 bp). The cycling conditions were 94°C melting, 60°C annealing, and 72°C extensions for 30 sec (30 cycles for eNOS and 28 cycles for GAPDH). The amplification conditions were optimized in preliminary studies to result in amplification within the linear range. PCR products were visualized on 1.5% agarose gels by ethidium bromide staining and gels were photographed under UV light. Relative gene expression was quantified by being densitometrically analyzed using image software. GAPDH transcript abundance was considered as an internal control to which eNOS transcript abundance was normalized.

### 2.8. Western Blot Analysis

Protein samples were prepared from cultured HUVECs with ice-cold cell protein lysates. Protein concentrations were measured using Bradford assay (Bio-rad protein assay kit). The protein samples (60 *μ*g) were denatured by boiling for 5 min, separated by 10% SDS-polyacrylamide gel, and then electroblotted at 4°C and transferred onto a polyvinylidene difluoride (PVDF) membrane (Bio-Rad). The membranes were blocked in 5% (w/v) nonfat milk for 2 h at room temperature and then incubated with rabbit polyclonal antibodies (eNOS, 1 : 800; Akt, 1 : 1000; AMPK, 1 : 1000; NOX4, 1 : 1000, Santa Cruz Biotechnology) with gentle agitation overnight at 4°C. The membranes were washed 3 times for 10 min each with 15 mL of TBST (10 mM Tris-HCl, 150 mM NaCl, and 0.1% (v/v) Tween-20) and then incubated with the second antibody (1 : 1000 goat Anti-rabbit IgG Horseradish Peroxidase Conjugate, Santa Cruz Biotechnology) at room temperature for 2 h. The protein was then visualized with enhanced chemiluminescence solution and X-ray film. To correct for differences in protein loading, the membranes were washed and reprobed with 1 : 2000 dilution goat polyclonal antibody to actin (Santa Cruz Biotechnology). An imaging densitometer was used to scan the protein bands and quantify them using the image analysis software.

### 2.9. Statistical Analysis

All data were expressed as mean ± S.E.M. The “*n*” denoted the sample size in each group. The statistical analyses were performed using one-way analysis of variance (ANOVA) followed by the Tukey post-hoc test. SPSS software (version 13.0 for Windows) was used for the statistical analysis. *P* < 0.05 was considered to be statistically significant.

## 3. Results

### 3.1. Effect of Berberine on HUVECs Viability

**Figure 1:**
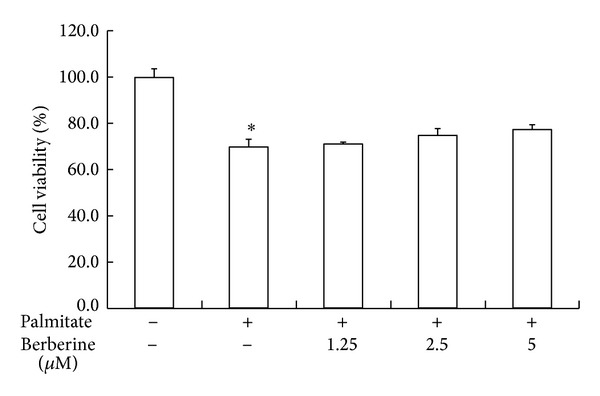
The effects of berberine on viability of HUVECs exposed to palmitate. HUVECs were cultured in RPMI-1640 containing 0.5 mmol/L palmitate and treated with 1.25, 2.5, and 5 *μ*mol/L berberine for 24 h. Cell viability was measured by MTT assay and normalized to cells incubated in control medium. Data were expressed as mean ± S.E.M. *P < 0.05 versus control.

HUVECs viability in the palmitate treated group fell to 70.03 ± 3.06% compared with that in the group without palmitate treatment. After berberine (1.25~5.0 *μ*mol/L) treatment in HUVECs, the cell viability was increased from 71.27 ± 3.05% to 77.27 ± 2.70% (Figure 1). Although this difference did not reach statistical significance, but the increased trend could be observed. In preliminary experiments, we found that higher concentrations of berberine (>5.0 *μ*mol/L) induced toxicities to HUVECs (data not shown). Accordingly, different concentrations of berberine (1.25~5.0 *μ*mol/L) were selected in the following studies.

### 3.2. Effect of Berberine on NO Levels in Cultured Medium of HUVECs

**Figure 2:**
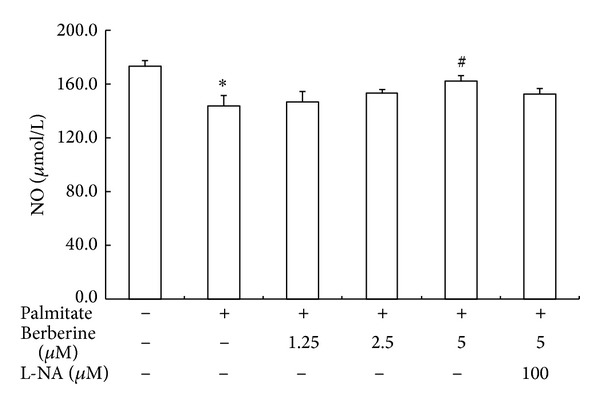
The effects of berberine on NO production in HUVECs exposed to palmitate. HUVECs were cultured in RPMI-1640 containing 0.5 mmol/L palmitate and treated with 1.25, 2.5, and 5 *μ*mol/L berberine for 24 h. The control group was not stimulated with 0.5 mmol/L palmitate. L-NA: N^G^-nitro-L-arginine. Data are expressed as mean ± S.E.M. *P < 0.05 versus control group, ^#^ *P* < 0.05 versus palmitate treated group.

HUVECs cultured with 0.5 mmol/L palmitate displayed a remarkable decrease in NO release compared with that of HUVECs without palmitate treatment (Figure 2). Berberine treatment significantly increased NO release as compared with untreated palmitate HUVECs. Berberine 5.0 *μ*mol/L had the strongest effect on NO release. eNOS inhibitor L-NA partially inhibited the effect of berberine on NO release. These results suggest that palmitate could reduce NO synthesis and release in cultured HUVECs, while berberine could significantly rescue the NO production which might be related to eNOS, the key enzyme of NO synthesis in endothelial cells.

### 3.3. Effect of Berberine on ROS in HUVECs

**Figure 3:**
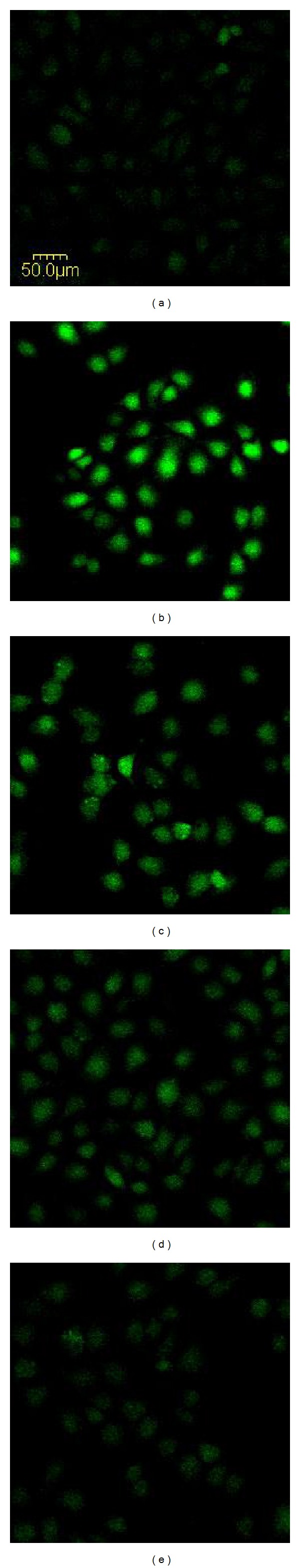
The effects of berberine on ROS in HUVECs exposed to palmitate. HUVECs were cultured in RPMI-1640 containing 0.5 mmol/L palmitate and treated with 1.25, 2.5, and 5 *μ*mol/L berberine for 12 h. HUVECs were labeled with DCFH-DA for 20 min and ROS generation was analyzed by fluorescence detection with Confocal microscopy at 200x. (a) HUVECs were cultured in RPMI 1640 without palmitate; (b) HUVECs were stimulated by 0.5 mmol/L palmitate; ((c), (d) and (e)) HUVECs were stimulated by 0.5 mmol/L palmitate and treated with 1.25, 2.5, and 5 *μ*mol/L berberine, respectively.

As shown in Figure 3, the green fluorescent intensity in HUVECs cultured with palmitate was significantly enhanced compared with that in the control group, which suggested that intracellular ROS levels in palmitate stimulated HUVECs were markedly increased. Berberine treatment decreased intracellular fluorescence intensity in a dose-dependent manner compared with palmitate group. These results indicate that palmitate could stimulate significantly the increase in ROS production and release from HUVECs, which might be related to cell injury caused by oxidative stress. It was believed that berberine treatment could reduce the production of ROS induced by high palmitate cultured HUVECs and play a protective effect on endothelial cells.

### 3.4. Effect of Berberine on eNOS mRNA Expression

**Figure 4:**
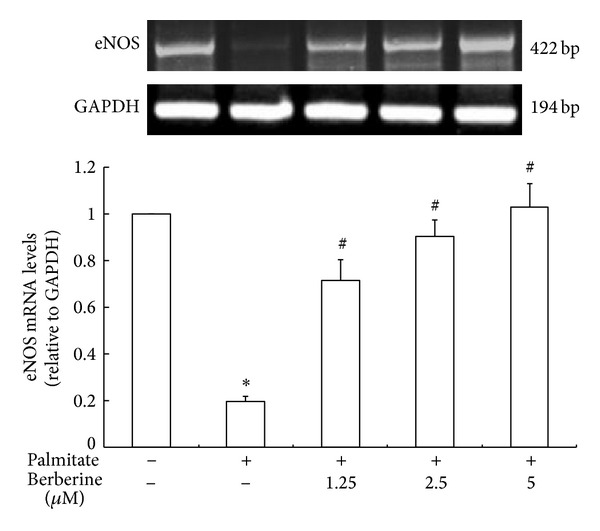
The effects of berberine on eNOS mRNA expression in HUVECs exposed to palmitate. HUVECs were cultured in RPMI-1640 containing 0.5 mmol/L palmitate and treated with 1.25, 2.5, and 5 *μ*mol/L berberine for 24 h. Total RNA was extracted and RT-PCR was performed. All values were normalized to 100% for the value of the control and were expressed as the percentage of the control. Data are mean ± S.E.M. The control group was not stimulated with 0.5 mmol/L palmitate. All presented results are representative of at least 3 independent experiments. *P < 0.05 versus control group. ^#^ *P* < 0.05 versus palmitate treated group.

Palmitate treatment significantly reduced eNOS mRNA expression in HUVECs versus control group (Figure 4), while intriguingly, berberine treatment can rescue the eNOS mRNA in palmitate-HUVECs in a dose-dependent manner (*P* < 0.05).

### 3.5. Effect of Berberine on Signaling of eNOS, Akt, and AMPK

**Figure 5:**
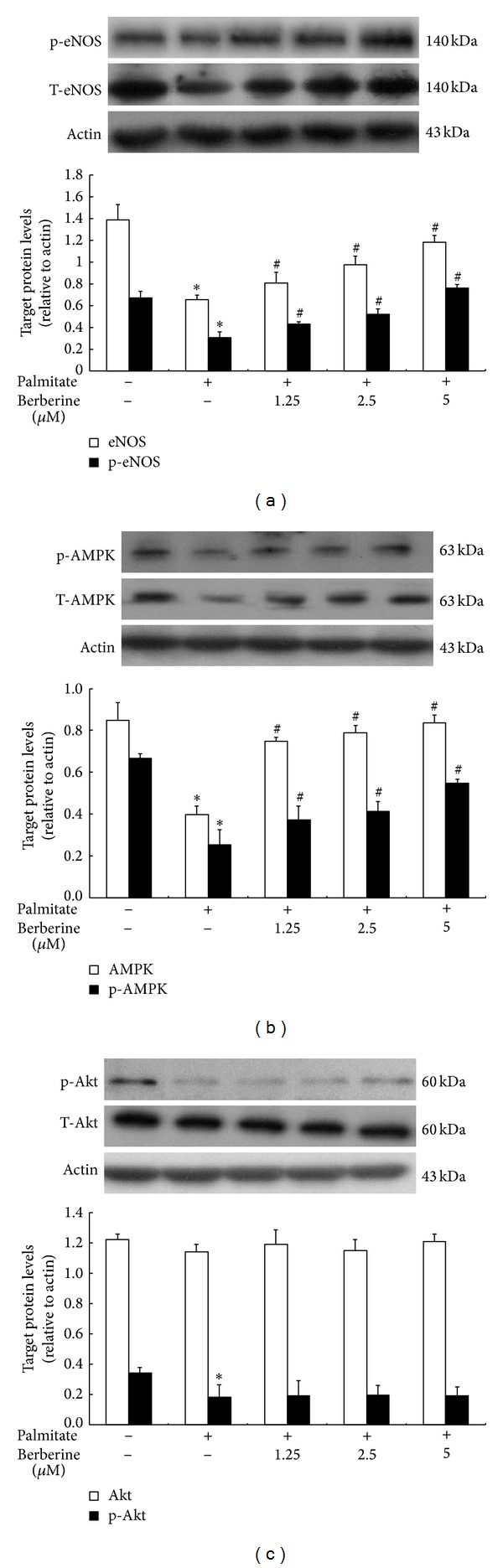
The effects of berberine on protein expression of eNOS, p-eNOS, Akt, p-Akt, AMPK, and p-AMPK in HUVECs exposed to palmitate. HUVECs were cultured in RPMI-1640 containing 0.5 mmol/L palmitate and treated with 1.25, 2.5, and 5 *μ*mol/L berberine for 24 h. Total protein was prepared and separated by SDS-PAGE. Expression and relative quantification of eNOS, Akt, and AMPK and phosphorylation of eNOS, Akt, and AMPK protein levels were expressed relative to the amount of actin. Control group was not stimulated with 0.5 mmol/L palmitate. Data are mean ± S.E.M. All presented results are representative of at least 3 independent experiments. *P < 0.05 versus control group, ^#^ *P* < 0.05 versus palmitate treated group.

To investigate whether berberine treatment could activate eNOS and its upstream kinase, Akt and AMPK in cultured HUVECs, eNOS, Akt, and AMPK protein expression were analyzed by western immunoblotting (Figure 5). The protein expression of eNOS and phosphorylation of eNOS were significantly reduced in HUVECs stimulated by palmitate compared to that of controls cells (Figure 5(a)). Berberine significantly increased eNOS and phosphorylation of eNOS protein expression. Compared with the group without palmitate, protein expression of total Akt had no significant change in HUVECs cultured with palmitate, while phosphorylation of Akt expression markedly decreased. Berberine treatment did not change the expression of Akt and p-Akt in HUVECs stimulated by palmitate (Figure 5(c)). However, the protein expression of AMPK, another upstream kinase of eNOS in endothelial cells, was significantly lowered in HUVECs cultured with palmitate compared with control group (without palmitate). Berberine increased not only the protein expression of total AMPK but also the phosphorylation of AMPK (Figure 5(b)). These results indicate that palmitate could downregulate eNOS expression in cultured HUVECs. Berberine treatment could reverse this change which might contribute to the activation of AMPK, promoting eNOS phosphorylation. While Akt/eNOS signaling pathway might not be involved.

### 3.6. Effect of Berberine on Protein Expression of NOX4

**Figure 6:**
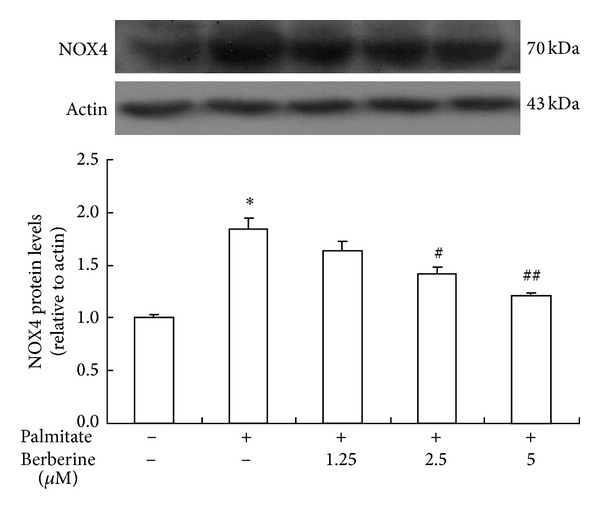
The effects of berberine on protein expression of NOX4 in HUVECs exposed to palmitate. HUVECs were cultured in RPMI-1640 containing 0.5 mmol/L palmitate and treated with 1.25, 2.5, and 5 *μ*mol/L berberine for 24 h. Total protein was prepared and separated by SDS-PAGE. Expression and relative quantification of NOX4 protein levels were expressed relative to the amount of actin. Control group was not stimulated with 0.5 mmol/L palmitate. Data are expressed as mean ± S.E.M. All presented results are representative of at least 3 independent experiments. *P < 0.01 versus control group, ^#^ *P* < 0.05 versus palmitate treated group, and ^##^ *P* < 0.01 versus palmitate treated group.

In contrast to eNOS expression, NOX4 protein expression, a main subunit of NADPH oxidase in vascular endothelium, was markedly enhanced in HUVECs stimulated by palmitate (Figure 6). Berberine treatment decreased the protein expression of NOX4 in HUVECs cultured with palmitate compared with control group (without palmitate). It suggests that berberine could reduce ROS levels by downregulating NOX4 expression.

## 4. Discussion

Increased oxidative stress and reduced NO bioavailability are important contributing factors and are closely related with inflammatory signaling pathways such as toll like receptor 4 signaling in the pathogenesis of endothelial dysfunction, hypertension, and cardiovascular and renal diseases [17]. Our previous studies showed that berberine restored endothelial vasodilation function under diabetic condition by enhancing NO bioavailability. In the present study, the direct effect of berberine on NO and ROS production was further observed in palmitate-induced endothelial injury of HUVECs. The results showed that the survival rate of HUVECs cultured with 0.5 mM palmitate for 24 h was significantly decreased. Moreover, berberine treatment significantly increased NO content in the supernatant of HUVECs cultured with palmitate. These results indicated that the protective effect of berberine on endothelial dysfunction induced by FFA might be associated with the elevation of NO levels.

eNOS is a key enzyme that produces NO in vascular endothelial cells. Studies on endothelial cells have demonstrated that FFA elevation in the culture medium can significantly decrease the activity of eNOS [18]. Palmitate and oleic acid could inhibit the phosphorylation of eNOS ser1177 sites, in turn reduce the NO production, which may contribute to endothelial dysfunction and the occurrence of atherosclerosis [18]. Healthy SD rats were administered fat emulsion and heparin in intravenous infusion; the elevated FFA level inhibited eNOS activity and expression and reduced endothelium-derived NO production in turn [19]. It has been reported that endothelium-derived NO production was mediated by Akt/eNOS signaling pathway [20]. In accordance with these findings, our results showed that palmitic acid significantly decreased the expression of eNOS in cultured HUVECs. Berberine treatment upregulated the expression of eNOS mRNA and total protein and promoted the phosphorylation of eNOS at ser1177 sites, thereby increasing the NO synthesis. Moreover, L-NA, one of eNOS inhibitors, partially attenuated NO production stimulated by berberine. Furthermore, it can be seen in the present study that palmitic acid remarkably reduced the phosphorylation of Akt in HUVECs. However, berberine did not affect the expression of Akt and its phosphorylation in HUVECs cultured with palmitic acid. It might indicate that berberine might exert its regulatory effect on eNOS activity by other ways.

Adenosine monophosphate-activated protein kinase (AMPK), as an intracellular energy receptor, has attracted more attention and become a new target for the treatment of diabetes and its cardiovascular complications due to its regulatory effect on endothelial cell function and energy homeostasis in recent years. AMPK plays an important role in regulating function of NO synthesis signaling pathways in endothelial cells. AMPK, an upstream kinase of eNOS, promotes the phosphorylation of eNOS Ser1177 site. AMPK can promote the formation of eNOS and HSP90 complex as well, thereby activating eNOS [17, 21]. Our results showed that berberine could significantly upregulate the expression levels of AMPK and p-AMPK protein of HUVECs cultured with palmitic acid, but had no effect on the expression of Akt and p-Akt protein. Accordingly, we speculated that the regulatory effect of berberine on eNOS activity and NO production might be related to the activation of AMPK partially.

Furthermore, endothelial dysfunction is also correlated with the production of ROS in vascular endothelial cells besides the decrease of NO production. It has been broadly accepted that elevated ROS levels are primarily derived from the action of NADPH oxidase (NOX). Activation of NOX could improve the ROS formation and contribute to endothelial dysfunction [22]. NOX4 is a subtype of NADPH oxidase expressed mainly in vascular endothelial cells, and it is the main source of O_2_^−^ production in the endothelial cells. In the present study, ROS production and NOX4 protein expression were measured in cultured HUVECs. Our results showed that palmitic acid significantly increased ROS production and the expression of NOX4 protein in cultured HUVECs. While berberine reduced ROS production and decreased protein expression of NOX4. These results are consistent with our previous findings from the diabetes animal models.

Several lines of studies indicated that AMPK is an important inhibitor of NADPH oxidase in cardiovascular cells. The activation of AMPK reduced ROS production by inhibiting the activity of NADPH oxidase and finally prevented the endothelial cell apoptosis induced by palmitic acid [23]. AMPK activators such as metformin may exert their cardiovascular protective function through NOX inhibition [24]. AMPK activation suppresses NOX activity may either block NOX phosphorylation and translocation to cell membrane or inactivate transcription factors including NF-*κ*B and STAT [25]. Therefore, berberine might prevent endothelial dysfunction from FFA-induced ROS generation by activation of AMPK. Taken together, berberine inhibits eNOS activation and NOX4-derived ROS accumulation in the HUVECs treated with FFA through AMPK activation, which may contribute to the protective effects of berberine on endothelial function. However, in our experiments, the specific inhibitor of AMPK was not used. It was still unknown whether the effect of berberine on the regulation of eNOS and NOX4 can be blocked by AMPK inhibitor, which should be explored to clarify the molecular mechanisms in the future studies. Furthermore, it has been reported that berberine could inhibit the TLR4-NF*κ*B pathway in LPS-induced intestinal injury in mice, a pathway involved in the impairment of eNOS expression and NO production. This might be another mechanism involved in the protective effect of berberine on endothelial dysfunction and still needs further investigation [26].

In summary, the present study investigated the protective effect of berberine on the vascular endothelial function in cell injury model induced by palmitic acid incubation, and revealed the underlying mechanism through which berberine can significantly ameliorate the endothelial dysfunction. Berberine could upregulate eNOS expression, enhance eNOS activity, and promote NO production. Meanwhile, berberine could downregulate NADPH oxidase expression and inhibit its activity to reduce ROS production and then inhibit NO inactivation as well. Thereby, berberine treatment could enhance the biological activity of NO to protect the vascular endothelial cell function. In our study, we also found that the regulatory effect of berberine on eNOS and NADPH oxidase activity may be related to the activation of AMPK. These results provide an important theoretical evidence for the application of berberine in the prevention and treatment of obesity, diabetes, and their cardiovascular complications.
